# On the Pathology and Characteristics of Insanity

**Published:** 1849-10-01

**Authors:** 


Art. III.—*Bibliothèque du Médecin Practicien; ou, Resumé General de tous les Ouvrages de Clinique Médicale et Chirurgicales, de toutes les Monographies, de tous les Mémoires de Médecine et de Chirurgie Practiques, Anciens et Modernes.* Publiés en France, et à l'Etranger par une Société de Médecins sous la direction du Docteur Farre, Auteur "Du Dictionnaire des Dictionnaires de Medicine," Redacteur-en-chef de la Gazette des Hopitaux : ouvrages adopté par l'Université. Tome Neuvième. Traité de Maladies du Cerveau, Maladies Mentales, Maladies Nerveuses. A Paris: chez J. B. Baillière;—à Londres: Baillière, 1849.

The Encyclopædias, Bibliothecas, and Dictionaries, which are published in France are universally esteemed works of high reputation; the most able and learned men of the age are contributors to them, and although the stream of knowledge flows rapidly onwards, the progress of science does not invalidate their authority. They are monuments of the epoch in which they appeared. The *Encyclopœdie de Médecine,* the *Bibliothèque Medicale,* the *Dictionnaire de Médecine,* are valuable and instructive records. Every article is a monograph from some distinguished pen ; they therefore remain standard works of reference, and each constitutes, as it were, a library in itself. To this high class of scientific literature the *Bibliothèque du Médecin Practicien* eminently belongs; it presents us with an elaborate account of the present state of knowledge in every department of medical science, and as the ninth volume, which has only just appeared in Paris, embraces the consideration of cerebral, mental, and nervous diseases, we hasten to lay before our readers a summary of that portion of its contents which relates especially to cerebral and mental pathology, and the general characteristics of insanity.

The volume comprehends, 1st, a description of those diseases of the brain which are characterized by anatomical lesions always, or nearly always, present; 2nd, an account of those diseases which have their probable seat in the nervous centres, and which leave behind them no anatomical lesion, or traces of lesions, so uncertain and slight as not to present us with any adequate cause for their existence; 3rd and lastly, mental diseases which in their anatomico-pathological relations come under these categories.

Here, then, we come at once to the *vexata questio,* whether organic lesions of the brain, either in the relation of cause or effect, are uniformly present in all cases of mental aberration? It is obvious, says the author of the article before us, that the simple functional derangement of any organ does not warrant us in expecting change of structure; hence he argues that hysteria, chorea, tetanus, epilepsy, and many idiopathic diseases leave no pathological appearances behind them; but then it is observed that insanity when prolonged, whether in an acute or chronic form, bears little or no analogy to these diseases, and it is reasonable to believe that in such cases the delicate texture of the brain will, however it may escape observation, be in some way or other organically affected. Were it not so, the disease might be pronounced purely mental, besides which experience proves that all functional disturbances lead to derangement of organic structure, and it becomes, therefore, a matter of profound interest and importance to determine what organic changes or lesions occur in the grey or white substance of the brain in cases of insanity which are well declared and of long standing.

Authorities on this subject, ancient and modern, in the pages before us are rapidly reviewed. Morgagni observed, in cases of insanity, induration and softening of the brain, and effusion into the ventricles. Greding noticed thickening of the cranial bones, fetidness and softening of the brain, and atrophy of the thalami nervorum opticorum. Haslam described adhesion of the pericranium to the bones, and their thickness and attenuation, as well as the consistency, and firmness or softness, of the cerebral substance. Prost endeavoured to prove that insanity was the effect of inflammation of the intestines. Rush ascribed its cause to the state of the blood-vessels in the brain; and next to him appeared two physicians of indisputable merit, Pinel and Esquirol, who maintained that anatomico-pathological causes have only a secondary influence in producing mental aberration. Thus Pinel positively affirms that cerebral lesions are the effect and not the cause of mental disease, and that they are often altogether wanting. So also Esquirol, after opening the head and examining many hundred brains, came to the conclusion that the morbid appearances he discovered were similar to those which he had found in persons who had died of other diseases; therefore he inferred that insanity was not to be ascribed to them, and although he has since somewhat modified this opinion, he has not described the character of those lesions which in such cases should be expected. M. Leuret, in an erudite and critical disquisition upon the alterations to which the brain is subject, asserts positively, that in a sufficient number of cases to determine the point under discussion, not the slightest morbid alteration has been perceptible. M. Baillarger and M. Brierre de Boismont, both high authorities, have adopted the same opinion. Other very eminent anatomists, however—MM. Foville, Delaye, Pinel, Grandchamp, Bayle, and Calmeil—have arrived at an opposite conclusion, and maintain that appreciable lesions may always be found in the brain, and that these bear a relation to and correspond with the character of the mental derangement. Here it may be well to remind our readers that the recent discoveries which have been made in the development and minute structure of the vesicular or grey matter have induced some physiologists to believe that this vesicular matter is the source of nervous power, and that mental operations are associated with or may be ascribed to certain changes which take place in the convoluted surface of the brain. This is the popular theory of the day. It therefore follows that in cases of insanity this vesicular structure, which is the presumed seat of the intellect, will undergo certain physical changes, and many who have adopted this hypothesis—which is, however, open to many serious objections—have not failed to discover minute lesions of this vesicular matter, to which all the phenomena of mental disease may be ascribed. These pathological appearances are as follows:—In the acute stage of insanity, the surface of this grey matter presents a certain degree of discoloration, which is more or less deeply tinted, slight effusion, general or partial redness, its consistency is sometimes increased and sometimes diminished, and the bloodvessels are frequently dilated; these morbid appearances decrease as we proceed from the anterior to the posterior region of the brain. In the chronic state of insanity, the grey substance detaches itself into two layers, with sometimes numerous little flesh-like processes. In some cases there is complete ramollissement. The volume of the convolutions may be diminished; in that case, there sometimes exists serous effusion, filling every depression and cavity; atrophy in the frontal region is more frequent than in the middle or posterior regions. The grey substance, too, although it may increase in consistency, sometimes entirely disappears. The cornu ammonis (hippocampus major) also presents a certain change in appearance. The white substance, or according to recent views, the fibrous or tubular portion of the brain, which is supposed to transmit the impressions originating in the vesicular matter, and connect the nervous centres with other organs and textures, is more exempt from any morbid appearance than the grey matter; but sometimes such alterations occur. It may appear to be injected or indurated, it may present a pearly colour, and sometimes the fibres will appear contracted and adhering among themselves. The morbid changes which the membranes of the brain undergo, such as vascularity, opacity, increased thickness, adhesions, &c., are well known; as are also those malformations which, by diminishing the capacity of the skull, interfere with the healthful development and action of the brain. The great objection, however, which may be urged against these different pathological appearances being esteemed significant of any particular form of mental derangement is that every one of these abnormal conditions has been found in the brains of persons who have died of other diseases, and who have never manifested a single symptom of insanity. Last year, M. Emery was called in to see a person of high rank, in Paris, who hitherto had enjoyed excellent health: "I feel very feeble," said the patient, as he supported himself against a piece of furniture in the room, and, notwithstanding the adoption of the most active means to support life, he almost instantly expired. The autopsy revealed a very extensive ramollissement in one of the cerebral hemispheres. In the present year, M. Rayer attended a young woman who for twelve days exhibited symptoms of cerebral affection; some considered it meningitis—others, acute delirium; but after death the brain was found to be only slightly injected. Injection, effusion, redness, or discoloration of the cerebral tissue may be the result of sanguineous congestion produced in the last moments of life by convulsions, epilepsy, apoplexy, interrupted circulation, and the difficulty of respiration, which may take place in the dying struggle. Then, again, proceeding to other well-marked organic changes, opacity, thickening, adhesion of the membranes, atrophy, and induration of the brain, as well as contraction and adhesion of the white fibres, with serous effusion have been considered to be the result of chronic inflammation; but all these alterations have been found in the brain of persons who never exhibited any signs of mental derangement, and whose delirium, when it existed, did not resemble insanity. M. Lélut discovered in the brains of twenty-five criminals, opacity and thickening of the arachnoid membrane. Such lesions are constantly found in extreme old age, although no symptoms whatever may have occurred at all analogous to or significant of mental derangement. The results of M. Lélut's induction upon the value which should be attached to alterations of the encephalon in acute delirium and in insanity are as follow:—"In acute delirium one half of the cases exhibit in the brain and its membranes, vestiges of inflammation; in acute mania, three at most out of twenty insane persons die from meningo-encephalic inflammation; and in the other cases, neither the brain nor its investing membranes present a single lesion explanatory of the maniacal symptoms. 2nd. In one half of the cases of chronic mania and simple dementia, no appreciable change is discoverable either in the brain or its membranes; in the other half, lesions are found. 3rd, and lastly, in none of these cases are the alterations which are presented either constant or exclusive, and the lesions which are met with from inflammation may be the results or effects of mental alienation, just as physical disorders result from nostalgia, succeeding to the grief which afflicts persons who are banished from their native country."

The absolute weight and specific gravity of the brain have also been subjects of investigation in France as in other countries. Meckel affirmed that the brain of the insane was lighter in weight than the brain of persons of sound mind. M. Leuret and Mitivié found that the mean specific gravity of the brain of an intelligent individual was 1·028, while that of an insane subject was 1·031. Parchappe came to the same conclusion. The weights of M. Lélut and M. Brierre de Boismont, present no appreciable difference. Last year (31st July, 1848), M. Parchappe presented the Academy of Sciences with a memoir on the gradual shrinking of the brain, consequent upon the progressive degradation of the intellect, in a state of insanity. It resulted from his observations in 498 cases, that the mean of the two categories—that is to say, acute and chronic insanity, presented a difference in weight equal to 89 *grammes* for the men and 85 *grammes* for the women, being a proportion equal to 77·1000 for the men and 67·1000 for the women. This difference is still more marked in that category which comprehends the mean of cases between the acute and last stage of chronic insanity, when the proportion reaches to 152 *grammes* for the men, or 114·100, and 135 *grammes*, or 100·1000, for the women.

There is, however, one form of mental disease in which anatomical lesions will be found both frequent and uniform, and that is in dementia, complicated with general paralysis. But with reference to the morbid appearances described by the authors referred to, they consist successively in adhesion of the membranes or chronic meningitis, diffuse encephalitis, a ramollissement of a layer of the grey matter, an adhesion between the white fibres, induration of the white substance, and ramollissement from the circumference to the central parts of the system. Lastly, to refer to the configuration of the skull, it is true that, in some cases of madness, it presents signal deformities; but it is also true, as was stated by M. Georget, that the heads of the insane are often found as well-shaped as those of sane and sensible people.

In the work under review, the inquiry is made whether the different forms of insanity which come under the designation of monomania, and which are characterized by the predominance of certain passions and desires, are accompanied by any corresponding organic development. Observation, says the writer, in cases of insanity, does not appear to justify the conclusions of Gall and Spurzheim, for he maintains patients are found exalted by ambition, fanaticism, love, &c., in whom there is no corresponding cranial development, and the region or site ascribed to these faculties is sometimes even unusually depressed. The editors of the *Bibliothèque* before us had under their care a distinguished literary man, who had the organ of language very prominent, but never manifested any taste for the study. There was the case also of the mad woman, mentioned by Lélut in the Salpêtrière, who, in the last stage of dementia, exhibited so strong a feeling for music, that she would repeat any air she incidentally heard, yet the organ of tune was extremely deficient. M. Combette presented to the institute the brain of a young girl, who had manifested intense sexual desire; and in her case the cerebellum was entirely wanting. The cast of the head of a young Indian girl was exhibited by M. Souty to the Academy of Medicine, which presented a very singular conformation; but no corresponding peculiarities were observed in her intellectual and moral character. We might also refer to the artificial flattening of the head of certain savage tribes, which does not impair the manifestations of the faculties ascribed to the subjacent organs. M. Lélut has endeavoured to establish, in a recent memoir on the subject, that the organ of destructiveness does not exist! The advocates of phrenology, it is true, affirm that excessive development of an organ is not necessary, because inflammation may give rise to a preternatural degree of its activity. The examination, says the writer, of the brain of persons who have died under monomania, has revealed the appearances which are common in general delirium. Sometimes the autopsy of persons, who have died under delirium, has presented circumscribed vestiges of inflammation, corresponding with the exact isolation of certain organs, which nevertheless manifested during life no characteristic activity of function. There are insane persons who occasionally exhibit protuberances of the supposed organs of veneration, acquisitiveness, amativeness, &c., who seek to conceal the exaggeration of their desires.

The great question of phrenology is of too important and too comprehensive a character to be thus cursorily discussed. The anatomico-pathological causes of insanity, neither the scalpel nor the microscope, have yet unveiled. What, then, is the nature of insanity?

In what does the disease itself consist? Pinel attributes mental alienation to an excessive exaltation of nervous energy. Cox ascribes it to an excessive afflux of blood through the brain. Foderé supposes that there is some alteration in the essence of the vital principle which he conceives to reside principally in the blood. Gall and Spurzheim presume that insanity consists in an inflammation—acute, then chronic—of the encephalon. According to Broussais, it is the result of irritation. Frank does not consider that insanity is a disease distinct from other diseases of the brain, but that it is developed in conjunction with an inflammatory, gastric, arthritic, rachitic, scrofulous, carcenomatous, or nervous diathesis. M. Delaye and Foville attribute mental alienation to inflammation of the superficial grey substance of the brain. Many authors imagine that mental derangement arises from sympathy with the abdominal viscera. Thus Dufour has endeavoured to prove that it depends upon an affection of the nervous plexuses in the abdomen, and that the brain is not implicated in the malady. Pinel states that it would appear in general that the cause of mental alienation is in the region of the stomach and of the intestines. Prost in particular conceives that the cause of insanity is to be found in the affection of the mucous membrane of the gastro-intestinal canal, and that its presence may often be traced to the existence of worms in the bowels. Esquirol affirms that it is derived from the different nervous centres of sensibility distributed through the different regions of the body, and not always from the brain. However, Gall, Georget, and many other eminent writers, have incontestably proved that the seat of insanity is in the brain, whatever may be its functional or organic condition; and most of the above views are reconcilable enough with the fact that the brain sympathizes with, and may be symptomatically affected by, various diseases of the intestinal canal, the uterus, and other organs. We must not, however, omit to notice the opinion of M. Leuret, and that also of the celebrated Heinroth, which may startle the spiritualist, and excite, perhaps, the risibility of the materialist. M. Leuret does not seek for the cause of insanity in any alterations of the cerebral structure. After examining and discussing various facts and well-known opinions, he comes to the conclusion that insanity may exist independently of any physical symptoms, and is an aberration of the mind itself—an affection purely moral. Hence it can be cured by moral treatment alone, and the method of cure should consist in substituting one mental impression for another mental impression—one passion for another passion. This doctrine that the mind only is the seat of the disease is also adopted by Heinroth, and involves many interesting and important points for consideration.

However doubtful may be the anatomico-pathological causes of insanity, and however obscure the seat of the disease, the secondary causes which modify and determine the forms of its development admit of clearer demonstration. These comprehend hereditary transmission, sex, age, temperament, civil state, professions, season, climate, atmospheric influence, and various moral causes connected with the intellect, affections, and passions, and the state of society as affected by political and religious commotions. It was observed by Esquirol that hereditary predisposition to insanity was six times more frequent among the rich than among the poor. In 1375 patients at Charenton, 337 were noted to be cases arising from hereditary transmission. In different parts of Europe the influence of this cause varies considerably. At Saint-You it prevails in 1 out of 6·55 cases; at Turin, 1 out of 8·32; at Palermo, 1 out of 15·30. According to M. Baillarger, who has paid great attention to the subject, out of 453 persons affected with insanity from direct hereditary transmission, 271 derived the disease from the mother, and 182 from the father. The conclusions which M. Baillarger arrives at are—1st, That insanity in the mother is a severer disease than in the father, not only because it occurs more frequently, but because she transmits it to a greater number of her children; 2nd, The disease transmitted direct from the mother is more likely to attack the girls than the boys, while transmitted from the father the boys are more likely to be attacked; 3rd, The mother may as well as the father transmit the disease to boys, but the attack is more to be feared in the girls when the mother is afflicted with it. The researches of M. Brierre de Boismont prove that the influence of hereditary transmission may be traced in about one-half of the lunatics he has seen in France, and in a great number examined by him in Italy, Germany, Belgium, Holland, and England. It may also be traced among the catholics in England, and the quakers; in most of the old Scotch families, among the Jews, and among princes. There is scarcely an old aristocratic family in France that does not number among its members an insane person, an idiot, or an epileptic. It was long since observed by the historians of Rome that the eternal city would have been destroyed in the third generation, had not the provinces, which were the veritable arteries of the empire, poured into it a supply of purer blood.

"We cannot," observes M. Brierre de Boismont, "too strongly recommend that every inquiry should be made concerning the sanity of the two families before any marriage is contracted. Insane, epileptic, scrofulous, consumptive persons, ought never to intermarry; nay, we will go further," he observes, "the married couple should not be both natives of the same town, and still less of the same capital—"*l'amelioration des races est à ce prix.*"

The proportion that exists between the sexes in cases of insanity, has been the subject of considerable research—some authors maintaining that females, and others that males, are more liable to the disease. In different localities, the proportion would appear to vary: thus, Pinel made the proportion between men and women so affected as 2·1; Esquirol, as 7·5; Earle makes a return from the United States of 4510 insane men, and 2480 insane women. Out of 76,526 cases, Esquirol found 38,825 males, and 37,701 females, giving a considerable preponderance on the women's side. When M. Brierre visited Italy in 1830, there were in the asylums there, 3442 patients, and adding to them 407 cases which he found in Sicily, he made a total of 3848 insane persons, of whom 1960 were men, and 1888 women. The preponderance here being in favour of the men. In the asylum of Sonnestein, at Pyrna, near Dresden, Dr. Klotz informed M. Brierre that he had 90 insane men to 60 women; in the hospital at Frankfort-on-the-Maine, he found 60 patients, and as many women affected as men. At St. Petersburg, on the 1st of January, 1832, there were 113 patients, of whom 54 were men, and 59 women. In the asylums in Belgium, Holland, and in England, M. Brierre, during his recent visit, found the proportion between the sexes to be nearly equal. Recently, Dr. Thurnam has also investigated the point, and from his statistical inquiries comes to the conclusion that men are more subject to insanity than women. The Report of the Commissioners in Lunacy, (London, 1845,) also on the 1st of January, 1844, return 36,044 insane males, and 31,832 females. The authors of the Compendium, upon the data afforded by numerous statistical returns, found that out of 60,318 cases, 31,580 were men, and 28,738 women. We must, therefore, come to the conclusion that men are more liable to insanity than women—an opinion which is entertained by M. Boutteville, and Parchappe, and other eminent physicians.

The age at which persons are most likely to be affected with insanity next claims attention. Infancy is not altogether exempt from this disease. M. Brierre states the case of a child ten years of age, who became maniacal after receiving a blow upon the head. He also gives that of a young lady, seven years of age, who saw angels in the skies, and was under various hallucinations. The insanity which occurs in early life most frequently assumes a maniacal or else a melancholic form. Two years ago, the public journals recorded ten suicides by children between nine and thirteen years of age. About the age of puberty, new desires and passions spring up, which become imperious, and frequently give rise to mental alienations. This is the epoch for erotic and religious aberration; and the desire to commit suicide is often at this age strongly manifested. Before, however, the age of twenty, insanity is certainly not of frequent occurrence; it is, indeed, scarcely to be anticipated before the brain has attained its maximum development. The following table, by Esquirol, exhibits the proportion of the sexes, and the ages of 12,869 patients observed by him in Paris:—

| | | | | | | | |
|---|---|---|---|---|---|---|---|
| Before 20 years, | 436 | men, | 348 | women, | 784 | total. |
| From 20 to 25 | ,, | 624 | ,, 563 | ,, | 1,187 | ,, |
| ,, 25 to 30 | ,, | 635 | ,, 727 | ,, | 1,362 | ,, |
| ,, 30 to 40 | ,, | 1,441 | ,, 1,607 | ,, | 3,048 | ,, |
| ,, 40 to 50 | ,, | 1,298 | ,, 1,479 | ,, | 2,777 | ,, |
| ,, 50 to 60 | ,, | 847 | ,, 954 | ,, | 1,801 | ,, |
| 60 and upwards | | 875 | ,, 1,035 | ,, | 1,910 | ,, |
| | | 6,156 | 6,713 | | 12,869 | |

In the following table, by Parchappe, which comprises 14,267 individuals, the ages and sexes are given thus:—

| | | | | | | |
|---|---|---|---|---|---|---|
| Below 20 years of age, | 469 men, | 518 women, | | 987 total. |
| From 20 to 29 years, | 1,451 | ,, 1,418 | ,, | 2,869 | ,, |
| ,, 30 to 39 ,, | 1,847 | ,, 1,782 | ,, | 3,429 | ,, |
| ,, 40 to 49 ,, | 1,340 | ,, 1,647 | ,, | 2,987 | ,, |
| ,, 50 to 59 ,, | 694 | ,, 1,110 | ,, | 1,804 | ,, |
| ,, 60 to 69 ,, | 519 | ,, 723 | ,, | 1,242 | ,, |
| ,, 70 to 79 ,, | 247 | ,, 448 | ,, | 695 | ,, |
| 80 and upwards | 27 | ,, 27 | ,, | 54 | ,, |
| | 6,594 | 7,673 | | 14,267 | |

The conclusions to be derived from these tables are sufficiently obvious: 1st, it is clear that the disease increases from twenty to thirty years of age; 2nd, from thirty to forty it attains its maximum; 3rd, from forty to fifty it diminishes, and after fifty years of age the diminution goes on still decreasing. The maximum admission for the two sexes is between thirty and thirty-nine years of age: the period from the age of twenty to thirty-nine furnishes the greater number of men, and from thirty to forty-nine the greatest number of women. From a table comprising 21,333 admissions, it is calculated that there is a decennnial progression and retrogression in the attacks of this disease. Thus—

| | | | | | |
|---|---|---|---|---|---|
| From 10 to 20 years of age, | we find | 1161 admissions, | or | 5·4 per cent. |
| ,, 20 to 30 | ,, | 5389 | ,, | or 25·3 | ,, |
| ,, 30 to 40 | ,, | 5621 | ,, | or 26·3 | ,, |
| ,, 40 to 50 | ,, | 4811 | ,, | or 22·6 | ,, |
| ,, 50 to 60 | ,, | 2715 | ,, | or 12·7 | ,, |
| ,, 60 to 70 | ,, | 1264 | ,, | or 5·9 | ,, |
| ,, 70 to 80 | ,, | 321 | ,, | or 1·6 | ,, |
| ,, 80 to 90 | ,, | 30 | ,, | or ·15 | ,, |

Hence it appears that the predisposition to insanity is not so great between twenty and thirty, as it is between thirty and forty years of age; and the decennial periods above forty mark its gradual retrogression. We must, however, remember, that climate and the different habits and manners of a country will very materially modify such returns: thus, in the United States, the greater proportion of cases occur between the age of twenty and thirty, which American physicians attribute to the nature of their institutions, and the circumstance of the young being sooner emancipated from their collegiate studies to participate in the business of the world. In Pennsylvania, out of 100 patients, 44·87 were attacked between twenty and thirty years of age, and only 18·9 between thirty and forty. So also in Ohio, the returns show, 43·97 between twenty and thirty, and 24·52 only between thirty and forty years of age. In France, however, it appears certain that persons are more liable to insanity between thirty and forty years of age than at any other period of life.

The influence of temperaments in modifying different forms of insanity, does not appear to be sufficiently well marked to lead to any result of practical importance; but it is observed, generally, that persons of a bilious temperament are predisposed to lypemania; those of a sanguine and nervous temperament to mania; and those who are of a lymphatic temperament to imbecility and dementia.

With reference to the civil state, it appears pretty well ascertained by the investigations of Esquirol and Parchappe, that insanity occurs more frequently among unmarried than among married persons. Celibacy in both sexes seems to have an equal influence in predisposing to the disease. Widows are more liable to insanity than widowers; and the married state protects men from it more than it does women.

The different seasons of the year are said to influence the development of insanity. At the Salpêtrière, it has been observed that the admissions are more numerous during the months of May, June, and July, than during the autumn and winter months. They diminish perceptibly between September and December, and still more in February and March. The maximum of admission is in June and July; the minimum, in January and February. It is also said that mania occurs more frequently in hot than in cold weather; and that damp and foggy seasons predispose to melancholia. Esquirol relates the case of a rich inhabitant of Holland who was subject to intermitting insanity, which attacked him regularly every autumn. He recommended him to travel into Italy, when this season approached, and by this means effected his cure. Epidemic insanity has been known to arise from certain conditions of the atmosphere: thus, Ramazzini relates, that during a very hot summer, the inhabitants of Abdera had, upon an extremely sultry day, assembled to witness a tragedy of Euripides, when all of a sudden a number of the spectators were seized with a cerebral fever, which terminated by profuse perspiration and nasal hæmorrhage about the seventeenth day; but during its access, they ran about the streets, shouting verses from Euripides, in a wild and frantic state. The soldiers who served under Napoleon in Egypt and Algiers were many of them seized with hallucinations, and some became maniacal, melancholic, and suicidal. In the disastrous retreat from Moscow, many officers, paralyzed with cold, became deranged, and were afterwards received into lunatic asylums. There would also appear to be certain local influences which favour the development of insanity. In Italy, a peculiar form of insanity occurs, which M. Brierre has described under the name of *Pellagra*, which is characterized by a strong desire to commit suicide, and an homicidal species of mania, in which individuals appear urged by an impulse they cannot resist, to kill their own children.

The moral causes of insanity are so various and so numerous, that we shall not here attempt to recount them; nor shall we enter into the question which appears to have been warmly contested by MM. Leuret and Parchappe, whether mental alienation increases as an effect of advancing civilization or not; we prefer dwelling upon the more practical portion of the volume before us, and proceed to consider the symptoms which characterize the invasion of the disease. These are often extremely obscure. The period of incubation may last for many days, weeks, or months, or even for a longer period. Pinel cites one in which this primordial stage lasted fifteen years. The attack will remain impending until on a sudden, more frequently during the night than the daytime, delirium will break out. This is, properly speaking, the period of its invasion. In some cases, however, insanity breaks out suddenly, the individual having up to the very moment of the outburst enjoyed the full possession of his intellectual faculties. An unexpected calamity, a political revolution, a reverse of fortune, at once destroys the equilibrium of the mental faculties; there may, however, be a derangement of the intellect so slight, as to render the recognition of the disease extremely doubtful and difficult. This state, which is not one of sound rationality nor yet of positive insanity, has been well described by M. Lelut. It is characterized by excessive sensibility, and among other symptoms may be observed, a difficulty of fixing the attention upon any given subject. It is in this, the early stage of insanity, that medical treatment will be found most availing; but it is always difficult to persuade the friends and relatives at this period that so terrible a malady is impending. The symptoms which now gradually develop themselves assume an almost infinite variety of character; it is indeed difficult to classify them. In the volume before us they are ranged under four heads: lesions of the intellect, lesions of sensibility, lesions of motility, and lesions of organic life. The intellectual faculties may individually or collectively be all more or less affected. The memory in particular, manifests remarkable peculiarities: it sometimes retains an extraordinary pertinacity in recalling past events, while those of recent occurrence are utterly obliterated. Occasionally, it would appear to be suspended at the very moment of the attack. Thus, Bergmann relates the case of an old man, aged ninety, who became insane when he was eighteen, and always believed that he remained at that age. The memory, however, of many remains perfectly unimpaired, and they have a vivid recollection of every incident that occurred during the attack, however maniacal they may have been. In other cases, however, it is utterly obliterated. A maniac who had been cured by prolonged baths and irrigations, told M. Rayer that the moment he was placed in the bath his memory left him. "I knew," said he, "that they put me into water for a long time, but all my sensations were confined to the impression that all around me was dark, then it became clearer, then the obscurity reappeared, after which I can remember nothing, it seemed like the tints of night, then day, and then evening." Sometimes, when a patient has been cured, the sight of certain objects will cause a relapse: thus, Professor Friedreich, of Wurtzburg, relates the case of a young man whose theological studies brought on a state of profound melancholia, for the cure of which he was removed to an asylum. He became convalescent, and upon his returning home, his father gave a *fête* in celebration of his recovery; upon leaving the table, he walked into the garden, and saw the asylum at a distance; instantly he became riveted, as it were, to the ground—his eyes fixed in the distance. In vain they attempted to remove him; he at length darted forwards towards the house in a state of mania, and seizing a leaden vase which was near him, and addressing his father in an infuriated tone, struck him on the head and killed him on the spot. The imagination is often affected in a very extraordinary manner. Insane persons have supposed themselves to be made of glass and of butter, and have taken extraordinary pains to prevent their being broken or melted. Everybody has probably read the case of the mad man reported in the *Revue Brittanique,* who imagined himself to be a teapot, and in order to simulate the handle, curved one arm round his body, resting his hand upon his hips, while to imitate the spout, he held the other out extended. This attitude he persisted in retaining. The grammarian Arthemidore was so frightened at the sight of a crocodile, that he became mad, and insisted that the animal had eaten away his foot and his left hand. In some cases, the intellectual faculties assume, during insanity, a preternatural activity, and talents are developed which were unknown in the normal state of the mind. Hence, some patients manifest an extraordinary taste for music, poetry, and painting; and sometimes they improvise verses with remarkable fluency and ability. Not unfrequently they take a particular dislike to persons they have never before seen, which occurs especially in dementia. It is also a curious fact, that upon recovery the disposition, taste, and habits often undergo a complete change. Another very remarkable circumstance which deserves the attention of psychologists, is the return of reason before death. M. Brierre relates the case of a young man who was for fifty-two years in his establishment in the Rue Neuve Saint Geneviève, and who had not spoken for thirty years. When perseveringly interrogated, he gave a kind of grunt and ran away. About fifteen days before his death, this patient, who had lost the habit of speaking for so long a time, and whose ideas were extremely circumscribed, recovered the use of his tongue, and answered perfectly well questions put to him. Very many such cases, even of a more remarkable kind, have come under our observation, and remind us of the beautifully-written dissertation of Aretæus on the clearing up of the mind before death. A very common symptom of insanity is the total perversion of the affections, and a feeling of antipathy and hatred against those who were previously beloved; but sometimes, on the other hand, the insane attach themselves pertinaciously to persons who were utter strangers to them. We remember an insane young woman who appeared to be devotedly attached to a sick friend; day and night for above a week she sat upon her bedside, ministering to her wants and comforts; but as soon as the object of her care died, she appeared to forget her entirely, and never spoke of her again. Many of the insane, however the intellect may be impaired, entertain a strong sense of right and wrong, justice and injustice; and their evidence upon matters of fact may often be implicitly relied upon.

Next to the lesions of intellect, the lesions of sensibility require to be noticed, and these are of two kinds—*special,* in which the functions of certain senses only are perverted, and *general,* in which the sensibility is generally impaired. Under the first category come hallucinations and illusions, and here it should be observed that M. Brierre insists upon a division of great importance,—viz., the separation of hallucinations into, 1st. *Physiological hallucinations,* and 2ndly, *Pathological hallucinations.* The former — physiological hallucinations — occur in dreams, and with children, and men absorbed and pre-occupied with a single idea or pursuit. In such cases, cerebral disease, we presume, will not be predicated. As examples of physiological hallucinations, we may refer to the history of Socrates, Plato, Luther, Joan of Arc, Ignatius Loyola, and many other celebrated enthusiasts. The latter species—pathological hallucinations – are very common among the insane who fancy they see strange figures, hear mysterious noises, and touch, taste, and smell things which exist only in their own imagination. Hallucinations frequently prompt to unusual, absurd, and sometimes dangerous actions,—even suicide and murder result from this perversion of the senses. Ravaillac, who assassinated Henri IV., imagined that he smelt fire and brimstone exhaling from his feet; he beheld figures dancing before him, and saw the host coming down through the air and sitting beside him; his voice resounded upon his ear like the sound of a trumpet, and one day he fancied that he saw a death's head upon a statue, all which, with many other hallucinations, induced him to believe that his victim had been sentenced to be damned, and that it was necessary that he should perish by his hand. The details of the cruel execution of this hallucinaut need not here be recapitulated. During the Huguenot civil war, similar hallucinations led, as probably they do in all religious wars and civil commotions, to the most hideous barbarities. Hallucinations may be confined to a single, or affect many senses. Illusions differ from hallucinations, inasmuch as they are based on some visible and sensible object, while hallucinations are purely imaginary. The square tower appears to be round—the river seems to fly—the shapes of bodies are transformed—the identity of individuals is mistaken —these, and a variety of other illusions, are constantly met with among the insane. The above examples illustrate the perversion of one or more special sensations; but the general sensibility of the body may be exalted, deranged, or abolished. There is an old soldier in the asylum of Saint-You, named Lambert, who believes that he was killed at the battle of Austerlitz. When he speaks of himself, he says, "This machine, which they thought to make like me, is very badly made." When he speaks of himself, he does not use the personal pronoun, *I*, but the demonstrative pronoun, *that*, as if speaking of some inanimate object. In him the general sensibility is extinguished, and he may be pinched or pricked without perceiving it. This loss of sensibility (excepting in cases of paralysis) is never permanent, it passes away, and then excessive sensibility sometimes supervenes. It is generally supposed that insane persons can bear with impunity the extremes of temperature; there are some, it is true, who can endure a very great degree of cold, but this is not frequently the case. In winter we find them always crowding round the fire, and exposure to cold constantly produces pains in the limbs, bowel complaints, and discoloration of the extremities. Unless kept warm, the feet will often exhibit symptoms of mortification; and occasionally great attention is required to keep up the circulation. The insensibility above described is sometimes local—*e. g.*, Dr. Burrows relates the case of a patient who put both his feet into a strong fire, which he made with the leaves of a book he had torn up, and who did not appear to suffer any pain from the flames. Another, without any apparent suffering, swallowed a glass of boiling water. An insane lady imagined that she was the daughter of the sun, and when it shone forth unclouded in the heavens, she would fix her eyes intently upon it for many hours, without being in the least dazzled; when clouds obscured its disk she became sad, and shut herself up in her apartment. The insensibility from the surface of the body would appear to extend to a certain degree internally—hence the intestinal canal is often insensible to the action of common doses of medicine. Mutzel relates the case of an insane patient, who required for an emetic seventeen grains of emetic tartar; the practice, however, of M. Brierre, is to give antimony and calomel in small doses, repeated several days in succession, which is attended with good effect.

Lesions of motility, or the functions of the muscular system, involve many important points for consideration in the diagnosis and treatment of insanity. Sometimes the insane appear to be endowed with preternatural strength—they walk to and fro with great vehemence, and gesticulate incessantly; at other times they are agitated, and their movements appear to be irregular and independent of their volition. There are patients who may be observed constantly throwing out their limbs, exercising the flexor and extensor muscles of the arms and legs, and striking the trunk of the body backwards against a post or a wall. These are frequently epileptics, and their disease is very often complicated with paralysis, the precursory symptoms of which are sometimes very obscure. The symptom which is generally first observed, is an unusual *embarras* of speech, the patient hesitates and stammers, and obviously has a difficulty in the articulation of certain letters and words. The tongue, when protruded, is tremulous; this hesitation of speech, however, will occasionally disappear for hours, particularly if he become excited, and he will then speak with great precipitation and rapidity; we may at the same time frequently observe an immobility and want of expression in the features,—the countenance is inexpressive, the muscles of the face seem relaxed. This hesitation and difficulty in speaking gradually increase, the words are no longer connected with each other, and a prolonged emphasis is placed upon each syllable. The articulation finally becomes unintelligible. In the meantime the functions of the muscular system fail, and here an interesting question suggests itself,—viz., whether this failure of muscular power proceeds from above downwards, or the reverse? M. Rodrigues has concluded, from his observations, that it begins with the tongue; thence extends to the superior limbs, and arrives gradually, or with more or less rapidity, at the lower extremities. M. de Crozant calls our attention to the insensibility of the skin, as indicating the approach of general paralysis. The liability of the two sexes to paralysis differs. Among 580 paralytic patients at Charenton, Saint-You, Antiquaille M. Rodrigues found 435 men, and 145 women, paralytic—that is to say, the proportion in favour of the men was as 4 to 1.

The diseases of organic life observed in insanity, comprehend those which are, under other circumstances, incident to the thoracic and abdominal viscera. The insane complain, upon the accession of the disease, of excessive thirst; sometimes they loathe their food, or else eat voraciously, and the tongue may be observed slightly coated of a white, or slightly yellowish, or brown colour. There is frequently pain in the epigastrium, and a general torpor of the digestive function, superinducing habitual and obstinate constipation. The heart is frequently, perhaps more frequently than any other vital organ, found diseased; ossification of the valves, and hypertrophy, are commonly met with. In many cases insanity is complicated with various neuralgic affections, and often with phthisis. The case is related of Madame B., who was suffering from phthisis when she became insane. During the several years that her insanity continued, the symptoms of the chest complaint disappeared. The disease of the mind being cured, the symptoms of phthisis again became developed; afterwards she relapsed into a state of insanity, and again the chest complaint was suspended. Eventually this lady recovered possession of her mental faculties, and the progress of phthisis then became so rapid, that she died soon after the recovery of her senses. This alternation of certain diseases with insanity has frequently been remarked, and only verifies the axiom of Hippocrates, that two distinct and acute diseases cannot co-exist in the same body.

When we consider the different forms under which insanity appears, it is obviously difficult to reduce them into any specific classification; hence the different divisions and sub-divisions which have been suggested, are all more or less unsatisfactory. The classification adopted by Esquirol, which he modified from Pinel, comprehends, 1. Lypemania, or Melancholia; 2. Monomania; 3. Mania; 4. Dementia; 5. Imbecilitas. This division, however, does not comprehend the "*délire aigu*," which establishes a transition between cerebral and mental affections; neither does it distinguish between hallucinations and illusions. The classification proposed by M. Brierre de Boismont, is considered to be the most comprehensive, and is as follows:—

GENERAL DIVISION.

| | |
|---|---|
| Loss of reason ................................ | Simple, accidental, congenital.<br>With lesion of sensibility.<br>With lesion of motility. |

SUBDIVISION.

*Form. Six Classes.*

| | | |
|---|---|---|
| 1st Class ................................ | Hallucinations.<br>Illusions. | |
| 2nd Class, Coherent general delirium ... | Acute delirium, (pyrexia.)<br>Mania, with or without furor. | |
| 3rd Class, Coherent delirium, gay, sad, or limited ................................ | to one idea (*rare*) .......... | Monomania. |
| | a small number of ideas ... | Oligomania. |
| | to one or more ideas, which change in the course of the disease | Trepomania. |
| | to a few ideas, without any external manifestation ...................... | Stupiditas. |
| 4th Class, Coherent delirium, general or limited, with or without disturbance of motility ...................... | Delirium of drunkards. | |
| 5th Class, Incoherent delirium ............. | Acute dementia.<br>Chronic dementia.<br>Progressive paralytic dementia.<br>Epileptic dementia.<br>Senile dementia. | |
| 6th Class, Weakness or absence of reason. | Imbecility.<br>Idiocy.<br>Cretinism. | |

The délire aigu, which forms the basis of this classification, is the phrenitis of the ancients, and the febrile delirium of the moderns; and is thus placed in accordance with the psychological phenomena exhibited in the different forms of insanity referred to. The subdivision of monomania suggests many very important considerations. M. Foville, and some other physicians, deny the existence of that species of monomania which is restricted to a single idea; but M. Baillarger has well argued that it is not to be assumed that the mind in such cases has not any other idea present to it; all that is meant is, that the reasoning faculties occupy themselves with one predominant idea, to which all other ideas are only accessory. He refers to the case of a patient, who, for upwards of twenty years, entertained the idea of killing *one person!* So also a magistrate of high probity and honour, conceived himself to be lost in consequence of having committed a criminal act, and so strongly was his mind possessed of this single idea, that in his more cheerful moments he could ridicule himself for the very act he believed that he had committed. In that species of monomania in which several ideas occupy the mind, (*oligomanie*), the patient is cheerful (*amēnomanié*), the eyes are bright, the countenance expressive of gaiety, and the conversation is thoughtless and unrestrained. In the opposite species of monomania, (*lypēmanie,*) the patient is melancholy; his countenance expresses inquietude and suspicion; he is taciturn and often suicidal. This species of monomania prevails, it is said, very much in the neighbourhood of Paris. There is a form of monomania which is not well understood, particularly in England; it is that species of insanity in which the reasoning faculties appear to be unimpaired, while the conduct of the individual is, in the highest degree, irrational. Pinel has designated this form of the disease, *manie raisonnante;* Brierre, *folie d'action;* Esquirol, *monomanie raisonnante;* and the late Dr. Pritchard, *moral insanity.* The individuals who are so affected will often talk in the most plausible manner, and explain their erratic conduct with so much ingenuity and address as to impose upon those who listen to them. In conversing with them it is impossible to detect any abberration of the intellectual faculties. They reason correctly, and often with more vivacity and ability than usual, particularly if they imagine that they are suspected and under any kind of surveillance; but the moment they are left to themselves, and believe they are not observed, they are guilty of great irregularity of conduct; they cannot rest in any one place; they annoy their companions, and excite one against the other by all kinds of falsehood and calumnies; they leave nothing about them untouched; they displace everything; and, should they be remonstrated with, they at once deny what they have done, or excuse and justify themselves with great tact; they never confess the truth, and have always a thousand good explanations to give for their conduct. Such patients are extremely troublesome and difficult to deal with. They frequently overwhelm the person in charge of them with compliments, and affect a tone of morality, sentiment, and religion; but the moment an opportunity occurs, they commit every kind of mischief which may, from the perversity of their disposition, suggest itself, and hence they become intolerable at home, or in other private families, and are apt even to destroy the discipline and subordination of the asylums into which they may be admitted. The symptoms of this form of disease are, a sudden change in the usual habits of living —caprice—versatility—estrangement and perversion of the moral affections—restlessness—and agitation. The intellectual faculties gradually become impaired, and a state of dementia ensues. The recognition of the disease itself requires experience, and the prognosis is generally unfavourable; so true it is, that the more intact the intellectual faculties remain, the greater always is the difficulty of cure.

Among the other forms of monomania, cases of homicidal and suicidal monomania are of frequent occurrence, but are often the result of what has appropriately been termed impulsive insanity. Hallucinauts are especially liable to commit either one or other of these acts, for they often hear voices commanding them sometimes to kill others, sometimes to kill themselves; and in many of these cases there exists previously no evidence whatever of mental derangement. Not unfrequently some peculiar fanatical notion suggests the fatal act; religious monomaniacs, therefore, are never safe. Pinel relates the case of a fanatic who conceived the idea that mankind should be regenerated by the baptism of blood; and under this delusion he cut the throats of all his children, and would have murdered his wife had she not effected her escape. Sixteen years afterwards, when a patient in the Bicêtre, he murdered two of his fellow-patients, and would have killed all the inmates in the hospital if his homicidal propensity had not been restrained. Instead of being impulsive, the homicidal act is sometimes premeditated; a fixed idea of vengeance occupies the mind until the favourable moment for consummating the act arrives. An insane patient having asked a female attendant in a private asylum for some money, was refused; he conceived immediately a feeling of resentment against the poor young woman, and having possessed himself of a piece of iron, sharpened the point of it, and for a fortnight carried the weapon concealed about his person, when suddenly a scream was heard, and it was found that he had stabbed her in the thigh, the sharp instrument having penetrated through her clothes, and divided the femoral artery. Upon suicidal monomania, many very interesting and curious facts are cited, some of which clearly prove that an hereditary predisposition to commit the act may exist.

It is stated that Gall used to relate the anecdote of a rich proprietor, who left a fortune of two millions between his seven children, who lived in the neighbourhood of Paris. They were very provident of their paternal inheritance, which some of them augmented. No misfortune happened to one of them. They enjoyed good health and an honourable position in society, but successively every one of these seven brothers committed suicide. He also knew a family in which the grandmother, sister, and mother, all committed suicide: the latter had two children, a daughter and son; the former was rescued from drowning herself, but the latter hung himself. It was the opinion of Gall that the cranial bones, in cases of suicides, present an increase in thickness and density; but this fact has not been verified. Cabanis imagined that the brains of suicides, and the insane generally, possess an excess of phosphorus, which is also not proved. Osiander ascribed the cause of this morbid propensity to lesions of the heart, and inflammation of the abdominal viscera. The perseverance of some patients to accomplish suicide almost exceeds belief; one patient attempted it successively forty-five times, and another, after having been for some months under the restraint of the camisole, the moment he was set free tried to dash his brains out against the wall. Some specific remedies have been recommended for this form of the disease, such as quinine, opium, musk. Avenbrugger advises that a seton should be placed over the region of the liver, and the patient be made to drink a great quantity of water. The actual cautery has also been prescribed, and blisters have certainly been found useful.

We must, however, here pause, as our limits preclude our giving, at present, a further analysis of this valuable work. In another article, we propose considering at length the views which are given upon the medical and moral treatment of insanity.

